# The 2016 ASE/EACVI recommendations may be able to more accurately identify patients at risk for diastolic dysfunction in living donor liver transplantation

**DOI:** 10.1371/journal.pone.0215603

**Published:** 2019-04-23

**Authors:** Jaesik Park, Jiyoung Lee, Ami Kwon, Ho Joong Choi, Hyun Sik Chung, Sang Hyun Hong, Chul Soo Park, Jong Ho Choi, Min Suk Chae

**Affiliations:** 1 Department of Anesthesiology and Pain Medicine, Seoul St. Mary’s Hospital, College of Medicine, The Catholic University of Korea, Seoul, Republic of Korea; 2 Department of Cardiology, Seoul St. Mary’s Hospital, College of Medicine, The Catholic University of Korea, Seoul, Republic of Korea; 3 Department of Surgery, Seoul St. Mary’s Hospital, College of Medicine, The Catholic University of Korea, Seoul, Republic of Korea; Imperial College Healthcare NHS Trust, UNITED KINGDOM

## Abstract

**Background:**

The aim of this study was to compare the prevalence of diastolic dysfunction between the 2016 American Society of Echocardiography (ASE)/European Association of Cardiovascular Imaging and 2009 ASE/European Association of Echocardiography recommendations in patients undergoing living-donor liver transplantation (LDLT).

**Patients and methods:**

A total of 312 adult patients who underwent LDLT at our hospital from January 2010 to December 2017 were retrospectively analyzed. Exclusion criteria were systolic dysfunction, arrhythmia, myocardial ischemia, and mitral or aortic valvular insufficiency.

**Results:**

The study population was largely male (68.3%), and the median age was 54 (49–59) years. The median model for end-stage liver disease score was 12 (6–22) points. A predominant difference in the prevalence rates of diastolic dysfunction was observed between the two recommendations. The prevalence rates of diastolic dysfunction and indeterminate diastolic function were lower according to the 2016 recommendations than the 2009 recommendations. The level of concordance between the two recommendations was poor. The proportion of patients with a high brain natriuretic peptide level (> 100 pg/mL) decreased significantly during surgery in the normal and indeterminate groups according to the 2009 recommendations; however, only the normal group showed an intraoperative decrease in the proportion according to the 2016 recommendations. Patients with diastolic dysfunction showed a poorer overall-survival rate than those with normal function according to both recommendations. However, there was a difference in the survival rate in the indeterminate group between the two recommendations. A significant difference in patient survival rate was observed between the dysfunction and indeterminate groups according to the 2009 recommendations; however, the difference was not significant in the 2016 recommendations.

**Conclusions:**

The 2016 classification may be better able to identify patients with a risk for diastolic dysfunction. Particularly, patients in the 2016 indeterminate group seemed to require a cardiac diastolic functional evaluation more frequently during and after surgery than those in the 2009 indeterminate group.

## Introduction

Diastolic dysfunction is a major component of cirrhotic cardiomyopathy and more frequently occurs than systolic dysfunction in patients with cirrhotic cardiomyopathy [1]. Diastolic dysfunction frequently leads to the development of heart failure and an increased risk for mortality [2,3]. Even in patients with mild diastolic dysfunction and a preserved ejection fraction (EF), there is an increased risk for cardiovascular events after surgery [4,5]. Because of peripheral vasodilation in patients with end-stage liver disease (ESLD), independent of etiology, latent cardiac dysfunction is masked at rest. An impairment of systolic or diastolic cardiac response is frequently present when a patient is stressed during and after surgery. As many as half of cirrhotic patients showed signs of diastolic dysfunction within the first week after liver transplantation (LT) [6,7]. More than 70% of patients who undergo LT suffer from one or more complications related to the heart after surgery [8].

Cardiac imaging has played an integral role in the assessment of LT candidates, and echocardiography, such as transthoracic (TTE) and/or transesophageal (TEE) echocardiography, has primarily been used in all liver transplant candidates to assess chamber size, hypertrophy, systolic and diastolic function, valvular function, and a left ventricular outflow tract obstruction [9,10]. However, no comprehensive study has fully performed cardiac diastolic assessments of patients scheduled for LT because the classification of diastolic function from a multiplicity of echocardiographic indices is difficult and there is a difference in hemodynamic condition between healthy subjects and patients with ESLD [11,12]. Particularly in South Korea, living donor liver transplantation (LDLT) has been more frequently performed than deceased donor liver transplantation [13]. Preoperative assessments of patients scheduled for LDLT are complex and comprehensive, and a tolerable cardiac condition is an important cornerstone related to improved outcomes [14]. Thus, there is a need to have better tools, including echocardiography, to identify patients at increased risk for diastolic dysfunction in LDLT.

Among cardiac biomarkers, brain natriuretic peptide (BNP) is mainly produced by elevated atrial or ventricular wall stretch and has been a promising factor to measure cardiac dysfunction. Accuracy in the risk stratification for heart failure is excellent with a cut-off value > 100 pg/mL [15]. Serum levels of BNP are prominently related to diastolic dysfunction determined by echocardiography in patients with a heart failure preserved ejection fraction (HFpEF) [16]. Compared with atrial natriuretic peptide, serum levels of BNP are more sensitive to identify the cardiac pathological progress, including heart structure and function [17]. In patients with ESLD, BNP plays a supportive role diagnosing cirrhotic cardiomyopathy, and a higher BNP level is strongly related to cardiac systolic or diastolic dysfunction and a poor survival rate [18,19].

Currently, the 2016 American Society of Echocardiography (ASE)/European Association of Cardiovascular Imaging (EACVI) recommendations have been proposed to more accurately diagnose cardiac diastolic dysfunction in the daily clinical setting than the previous 2009 ASE/European Association of Echocardiography (EAE) recommendations [20,21]. However, previous community-based studies suggested a significant difference in the prevalence of diastolic dysfunction between the 2016 and 2009 recommendations [22,23]. The aim of this study was to compare the prevalence of diastolic dysfunction between the 2016 and 2009 recommendations in patients undergoing living-donor liver transplantation (LDLT). Additionally, we analyzed the changes in serum levels of BNPs during the surgery, and the short- and long-term outcomes after the surgery.

## Patients and methods

### Ethical consideration

This study was approved by the Institutional Review Board of Seoul St. Mary’s Hospital Ethics Committee (KC18RESI0205) according to the Declaration of Helsinki, and the requirement for informed consent was waived due to the retrospective study design.

### Study population

A total of 366 adult patients (≥ 19 years) underwent LDLT at the Seoul St. Mary’s Hospital from January 2010 to December 2017. The data were retrospectively reviewed using the medical records system in our hospital. Exclusion criteria for this study were: systolic dysfunction (i.e., EF < 50%) (n = 3), arrhythmia (i.e., atrial fibrillation or flutter and bundle branch block) (n = 23), history of myocardial ischemia (i.e., percutaneous or surgical myocardial revascularization) (n = 5), mitral or aortic valvular insufficiency (i.e., more than mild) (n = 10), and missing or inappropriate data (n = 54), including transthoracic echocardiography (TTE) findings (n = 13). In total, 312 adult patients were analyzed.

### Patient management

According to our hospital LDLT protocol that was described previously in detail [24,25], the piggyback surgical procedure was applied using the right lobe from the living donor that was larger than 40% of the recipient’s standard liver volume or 0.8% of the recipient’s body weight [26]. After completing the hepatic vascular anastomosis, intact hepatic circulation was demonstrated using Doppler ultrasonography. Balanced anesthesia care was meticulously performed under multiple vital monitoring with serial laboratory assessments. A triple-drug regimen (i.e., tacrolimus, mycophenolate mofetil, and prednisolone) with basiliximab (i.e., interleukin-2 receptor antagonist) was administered for immunosuppression during the perioperative period and gradually tapered after surgery.

### Echocardiographic evaluation

Cardiac function was carefully measured preoperatively to prevent foreshortening the atrium and ventricle and averaged over three serial cardiac cycles using two transthoracic echocardiographic devices (GE Healthcare, Vivid E9, Milwaukee, WI, USA), (PHILIPS Healthcare, iE33, Durham, NC, USA) by experienced cardiologists. Left ventricular (LV) EF was calculated using the biplane method (i.e., modified Simpson’s rule) on apical four- and two-chambered views. Lateral or septal mitral annulus velocity (i.e., e’ wave) was evaluated using tissue Doppler imaging for LV diastolic function measured on apical views. Mitral inflow velocities (i.e., E and A waves) and deceleration time were measured using pulse-wave Doppler imaging. Peak tricuspid regurgitation (TR) velocity was measured using continuous-wave Doppler imaging. Left atrial (LA) volume was measured using the biplane method (modified Simpson’s rule) on apical views and indexed to body surface area (BSA) (i.e., LV volume/BSA = left atrial volume index [LAVI]). LV hypertrophy type was classified by the LV mass index and relative wall thickness [27,28].

### Classification of diastolic function

The prevalence of diastolic function was evaluated according to the 2016 and 2009 recommendations [20,21]. According to the four diastolic parameters (i.e., average E/e’ > 14; septal e’ velocity < 7 cm/s or lateral e’ velocity < 10 cm/s; peak TR velocity > 2.8 m/s; LAVI > 34 mL/m^2^) in the 2016 recommendations, the patients were classified into three groups, including the normal diastolic function group (i.e., normal group), indeterminate function group (i.e., indeterminate group), and the diastolic dysfunction group (i.e., dysfunction group). Normal diastolic function was determined as the available diastolic parameters < 50%; diastolic dysfunction was determined as the available diastolic parameters > 50%; and indeterminate function was determined as the available diastolic parameters of 50%. In the 2009 recommendations, septal e’ velocity < 8 cm/s or lateral e’ velocity < 10 cm/s and LAVI ≥ 34 mL/m^2^ were used to evaluate diastolic function. The normal group was defined as no available diastolic parameters, and the diastolic group was defined as all available diastolic parameters. When it was not possible to determine diastolic function because of a discrepancy in diastolic parameters, the subjects were placed in the indeterminate group.

### BNP measurement

Intraoperative BNP levels were evaluated three times, such as in the preanhepatic phase (i.e., immediately after inducing anesthesia); in the anhepatic phase (i.e., starting at the portal venous anastomosis); and in the neohepatic phase (i.e., starting at peritoneal closure) [24,29]. BNP levels were investigated via enzyme-linked immunosorbent assay using a Siemens ADVIA Centaur (Siemens Healthcare Diagnostics, Erlangen, Germany). The analytical process was a fully automated two-site sandwich immunoassay using direct chemiluminescent technology. The detection range was 2–5,000 pg/mL according to the manufacturer. BNP levels were classified into high *vs*. low based on a cut-off value of 100 pg/mL [15].

### Clinical variables

Preoperative factors included age, sex, body mass index (BMI), diabetes mellitus, hypertension, model for end-stage liver disease (MELD) score, etiology, hepatic decompression signs, QTc prolongation (> 440 ms) [30], and laboratory values (i.e., hematocrit, sodium, platelet count, and albumin). Intraoperative factors included surgical duration, postreperfusion syndrome (PRS) [31], strong vasopressor administration (i.e., epinephrine or norepinephrine), and total amount of blood product transfused (i.e., packed red blood cells [PRBCs] and fresh frozen plasma [FFP]). Liver graft factors included graft volume and total graft ischemic time. Postoperative outcomes included total hospital and intensive care unit (ICU) stays, overt heart failure (i.e., heart failure reduced ejection fraction [HFrEF] ≤ 40%) [32], supportive devices (i.e., mechanical ventilation and continuous renal replacement therapy [CRRT]) in ICU, early allograft dysfunction (EAD) [33], and acute kidney injury (AKI) [34], and overall patient survival rate.

### Statistical analysis

The distribution of continuous data was assessed using the Shapiro–Wilk test. The values are expressed as medians (interquartile range) and numbers (proportion). The perioperative data were compared between the normal, indeterminate, and dysfunction groups using the Kruskal–Wallis test with the Mann–Whitney *U*-test as a *post-hoc* test. The categorical data were evaluated using χ^2^ or Fisher’s exact tests, as appropriate. The test for trends was conducted using a linear-by-linear association method. Concordance in the prevalence of diastolic dysfunction according to the 2016 ASE/EACVI and 2009 ASE/EAE recommendations was evaluated using a Cohen’s kappa coefficient with 95% confidence intervals (CIs). The strength of concordance was classified into poor (kappa < 0.2); fair (kappa range 0.21–0.4); moderate (kappa range 0.41–0.6); good (kappa range 0.61–0.8); and very good (0.81–1.0). Intraoperative changes in the proportions of patients with high BNP levels (≥ 100 pg/mL) were analyzed using Cochran’s Q test with the McNemar *post-hoc* test. Overall patient survival during the follow-up period was analyzed using the Kaplan–Meier test, and compared between the normal, indeterminate, and dysfunction groups using the log-rank test. Multiple comparisons were adjusted with Bonferroni’s method. A *p*-value<0.05 was considered significant. Statistical analyses were performed using SPSS for Windows (*ver*. 24; SPSS Inc., Chicago, IL, USA) and MedCalc for Windows (*ver*. 11.0; MedCalc Software, Ostend, Belgium).

## Results

### Clinical demographic characteristics

Table 1 shows that the study population was largely male (68.3%) and median age was 54 (49–59) years. The median BMI was 24.1 (22.1–26.8) kg/m^2^. The incidence rates of diabetes mellitus and high blood pressure were 25.0% (n = 78) and 19.6% (n = 61), respectively. The etiologies for LDLT were hepatitis B (54.5%), alcohol (20.8%), hepatitis C (9.3%), autoimmune (4.5%), hepatitis A (2.2%), drug or toxin (1.6%), and cryptogenic hepatitis (7.1%). 146 patients (46.8%) underwent LDLT because of hepatocellular carcinoma (HCC). The median MELD score was 12 (6–22) points, and hepatic decompensation signs were encephalopathy (West Haven grade III or IV) (5.3%) [35], esophageal varices (26.3%), ascites (47.9%), and hepatorenal syndrome (16.0%). The median QTc interval on electrocardiography (ECG) was 449 (427–469) ms. The median hematocrit and sodium levels were 29.4 (24.9–35.0) % and 140 (135–142) mEq/L, respectively. The median platelet and albumin levels were 65.5 (46.3–103.0) × 10^9^/L and 3.0 (2.6–3.6) g/dL, respectively. The median surgical duration was 490 (445–540) min. A total of 239 patients (76.6%) developed PRS, and 215 patients (69.4%) were required to receive a strong vasopressor (i.e., norepinephrine or epinephrine) to stabilize their hemodynamics. The median PRBC and FFP requirements were 7 (4–12) and 6 (4–10) units, respectively. The median graft volume was 891.9 (760.4–1,038.9) mL, and the median total graft ischemic time was 92 (69–129) min.

**Table 1:** Clinical characteristics of patients undergoing living-donor liver transplantation.

Clinical characteristics	
n = 312	
***Preoperative finding***	
Age (years)	54 (49–59)
Sex (male)	213 (68.3%)
Body mass index (kg/m^2^)	24.1 (22.1–26.8)
Diabetes mellitus	78 (25.0%)
Hypertension	61 (19.6%)
Model for end-stage liver disease score (points)	12 (6–22)
Etiology	
Alcohol	65 (20.8%)
Hepatitis A	7 (2.2%)
Hepatitis B	170 (54.5%)
Hepatitis C	29 (9.3%)
Autoimmune	14 (4.5%)
Drug or toxin	5 (1.6%)
Cryptogenic hepatitis	22 (7.1%)
Hepatocellular carcinoma	146 (46.8%)
Hepatic decompression sign	
Encephalopathy (West Haven III or IV)	16 (5.3%)
Esophageal varix	82 (26.3%)
Ascites	149 (47.9%)
Hepatorenal syndrome	50 (16.0%)
QTc prolongation on electrocardiogram (ms)	449 (427–469)
Laboratory values	
Hematocrit (%)	29.4 (24.9–35.0)
Sodium (mEq/L)	140 (135–142)
Platelet count (× 10^9^/L)	65.5 (46.3–103.0)
Albumin (g/dL)	3.0 (2.6–3.6)
***Intraoperative finding***	
Surgical duration (min)	490 (445–540)
Postreperfusion syndrome	239 (76.6%)
Strong vasopressor administration	215 (69.4%)
Total amount of blood product transfusion (units)	
Packed red blood cells	7 (4–12)
Fresh frozen plasma	6 (4–10)
***Liver graft finding***	
Graft volume (mL)	891.9 (760.4–1,038.9)
Total graft ischemic time (min)	92 (69–129)

**NOTE**: Values are medians (interquartile range) and numbers (proportion).

### Transthoracic echocardiography according to the 2016 ASE/EACVI recommendations

Four diastolic dysfunction parameters in the 2016 recommendations (i.e., average E/e’ > 14; septal e’ velocity < 7 cm/s or lateral e’ velocity < 10 cm/s; TR velocity > 2.8 m/s; and LAVI > 34 mL/m^2^) were evaluated in our study cohort (Table 2): average E/e’ > 14 was present in 5.4% (n = 17); septal e’ velocity < 7 cm/s or lateral e’ velocity < 10 cm/s in 30.4% (n = 95); TR velocity > 2.8 m/s in 8.7% (n = 27); and LAVI > 34 mL/m^2^ in 41.7% (n = 130). The prevalence of these diastolic parameters increased from the normal group to the indeterminate and dysfunction groups. The mitral E/A ratio was lower in the indeterminate group and higher in the dysfunction group than in the normal group. Deceleration time was shorter in the dysfunction group than in the normal group. LVEF was comparable between the three groups. The proportion of abnormal chamber geometry (i.e., eccentric hypertrophy, concentric remodeling and hypertrophy) was higher in the indeterminate and dysfunction groups than in the normal group.

**Table 2:** Comparison of preoperative transthoracic echocardiographic characteristics according to diastolic function in the 2016 ASE/EACVI recommendations in patients undergoing living-donor liver transplantation.

Diastolic function group	Normal	Indeterminate	Dysfunction	*p*
n = 312	260 (83.3%)	40 (12.8%)	12 (3.8%)	
**Diastolic parameter** **in the 2016 ASE/EACVI recommendations**				
Average E/e’ > 14	3 (1.2%)	4 (10.0%)^††^	10 (83.3%)^†††^	< 0.001
Septal e’ velocity < 7 cm/s or Lateral e’ velocity < 10 cm/s	54 (20.8%)	29 (72.5%)^†††^	12 (100.0%)^†††^	< 0.001
TR velocity > 2.8 m/s	9 (3.5%)	13 (32.5%)^†††^	5 (41.7%)^†††^	< 0.001
LA volume index > 34 mL/m^2^	83 (31.9%)	36 (90.0%)^†††^	11 (91.7%)^†††^	< 0.001
**Additional diastolic parameters**				
Mitral E/A ratio	1.0 (0.8–1.3)	0.9 (0.7–1.3)^†^	1.8 (1.5–1.9)^†††^	< 0.001
Mitral deceleration time (ms)	202 (173–240)	208 (177–225)	130 (114–169)^†††^	< 0.001
Left ventricular ejection fraction (%)	64 (62–67)	65 (61–68)	64 (62–67)	0.829
**Left ventricular chamber quantification (%)**^‡^				< 0.001
Normal Geometry	168 (64.6%)	17 (42.5%)^††^	3 (25.0%)^†^	
Eccentric hypertrophy	42 (16.2%)	14 (35.0%)^††^	4 (33.3%)	
Concentric remodeling	34 (13.1%)	5 (12.5%)	1 (8.3%)	
Concentric hypertrophy	16 (6.2%)	4 (10.0%)	4 (33.3%)^††^	

**NOTE:** Values are medians (interquartile range) and numbers (proportion).

^‡^Left ventricular chamber quantification was classified according to the left ventricular mas index (> 115 g/m2 in males and > 95 g/m2 in female) and relative wall thickness (> 0.42).

^†^*p* value < 0.05 based on the normal group value

^††^*p* value < 0.01 based on the normal group value

^†††^*p* value < 0.001 based on the normal group value

### Comparison of prevalence of diastolic dysfunction between the 2016 ASE/EACVI and 2009 ASE/EAE recommendations

**Fig 1:**
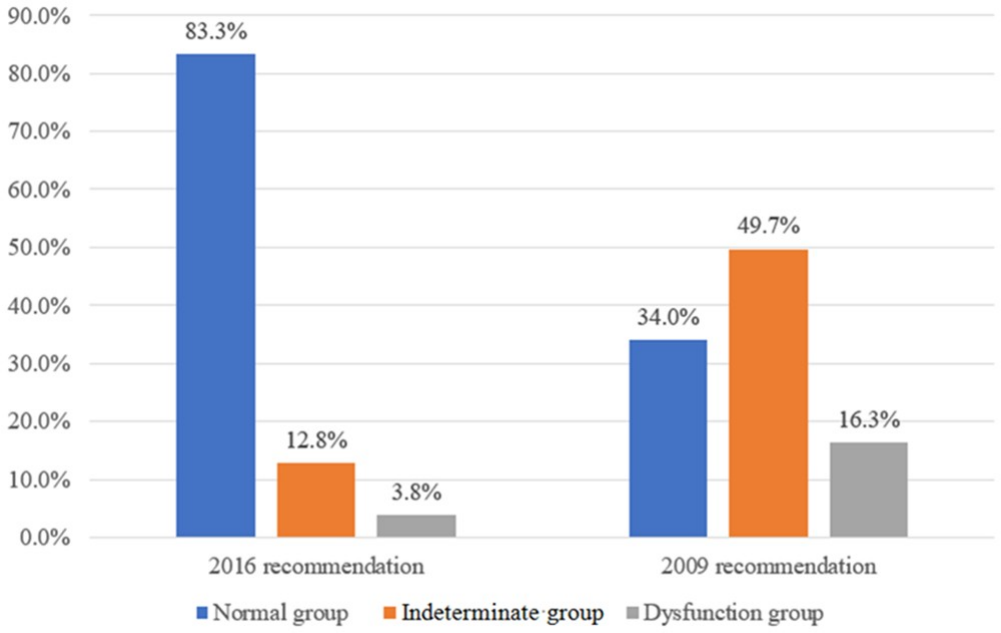
Prevalence of diastolic dysfunction according to each classification in patients who underwent living-donor liver transplantation.

According to the 2016 recommendations (Fig 1), 260 patients (83.3%) had normal diastolic function; 40 patients (12.5%) had indeterminate diastolic function; and 12 patients (3.8%) had diastolic dysfunction. However, according to the 2009 recommendations, 106 patients (34.0%) had normal diastolic function; 155 patients (49.7%) had indeterminate diastolic function; and 51 patients (16.3%) had diastolic dysfunction.

All patients (n = 106) with normal diastolic function according to the 2009 recommendations were in the normal diastolic function group according to the 2016 recommendations (Table 3). However, 14 patients with indeterminate diastolic function in the 2009 recommendations (9.0%) were in the indeterminate diastolic function group according to the 2016 recommendations; 140 patients with indeterminate diastolic function in the 2009 recommendations (90.3%) were in the normal diastolic function group in the 2016 recommendations; and one patient with indeterminate diastolic function in the 2009 recommendations (0.6%) was in the diastolic dysfunction group in the 2016 recommendations. Additionally, 11 patients with diastolic dysfunction according to the 2009 recommendations (21.6%) were in the diastolic dysfunction group in the 2016 recommendations; 14 patients with diastolic dysfunction in the 2009 recommendations (27.5%) were in the normal diastolic function group in the 2016 recommendations; and 26 patients with diastolic dysfunction in the 2009 recommendations (51.0%) were in the indeterminate diastolic function group in the 2016 recommendations. Therefore, concordance in the prevalence of diastolic dysfunction between the 2009 and 2016 recommendations was poor (Cohen’s kappa coefficient = 0.103; 95% CI = 0.019–0.188) in patients undergoing LDLT.

**Table 3:** Concordance in diastolic dysfunction prevalence according to the 2009 ASE/EAE recommendations and the 2016 ASE/EACVI recommendations.

	**2009 recommendation**		
	Normal group	Indeterminate group	Dysfunction group
**2016 recommendation**			
Normal group	106 (100.0%)	140 (90.3%)	14 (27.5%)
Indeterminate group	0 (0.0%)	14 (9.0%)	26 (51.0%)
Dysfunction group	0 (0.0%)	1 (0.6%)	11 (21.6%)
Total (n = 312)	106 (34.0%)	155 (49.7%)	51 (16.3%)

**NOTE:** Values are numbers and proportions.

### Comparison of preoperative MELD score according to diastology in the 2016 ASE/EACVI and the 2009 ASE/EAE recommendations

Higher MELD score was moderately correlated with the degree of diastolic function in the 2016 recommendations, but the correlation between MELD score and diastolic function in the 2009 recommendation was weak (S1 Table). In both recommendations, the proportion of patients with a high MELD score (> 16 points) increased significantly in accordance with degree of diastolic function (S2 Table).

### Comparison of intraoperative changes in the proportion of patients with a high serum levels of BNP (≥ 100 pg/mL) between the 2016 ASE/EACVI and the 2009 ASE/EAE recommendations

Table 4 shows the intraoperative serial changes in the proportion of patients with a high serum levels of BNP (>100 pg/mL) at the preanhepatic, anhepatic, and neohepatic phases in each group, and differences in the proportion of high BNP level at each phase among the three groups. According to the 2016 recommendations, the proportion of patients with a high BNP level decreased significantly from the preanhepatic phase to the anhepatic and neohepatic phase in the normal group, but not in either the indeterminate or dysfunction groups. According to the 2009 recommendations, the proportion of patients with a high BNP level decreased significantly through the surgical phases in both the normal and indeterminate groups, but not in the dysfunction group. According to the 2016 recommendations, the proportion of patients with a high BNP level at the neohepatic phase was higher in the indeterminate and dysfunction groups than in the normal group; however, the proportion was only higher in the dysfunction group than in the normal group according to the 2009 recommendations.

**Table 4:** Intraoperative change in the proportion of patients with a high serum brain natriuretic peptide level (>100 pg/mL) between the normal diastolic, indeterminate, and diastolic dysfunction groups according to the 2016 and 2009 recommendations during living-donor liver transplantation.

	2016 recommendation				2009 recommendation			
Diastolic function group	Normal	Indeterminate	Dysfunction	*p*	Normal	Indeterminate	Dysfunction	*p*
n = 312	260 (83.3%)	40 (12.8%)	12 (3.8%)		106 (34.0%)	155 (49.7%)	51 (16.3%)	
Serum brain natriuretic peptide level								
**Preanhepatic phase**								
≤100 pg/mL	125 (48.1%)	8 (20.0%)	2 (16.7%)	0.001	51 (48.1%)	70 (45.2%)	14 (27.5%)	0.04
>100 pg/mL	135 (51.9%)	32 (80.0%)^††^	10 (83.3%)^†^		55 (51.9%)	85 (54.8%)	37 (72.5%)^†^	
**Anhepatic phase**								
≤100 pg/mL	165 (63.5%)	12 (30.0%)	5 (41.7%)	<0.001	82 (77.4%)	81 (52.3%)	19 (37.3%)	< 0.001
>100 pg/mL	95 (36.5%)***	28 (70.0%)^†††^	7 (58.3%)		24 (22.6%)***	74 (47.7%)*^,^^†††^	32 (62.7%)^†††^	
**Neohepatic phase**								
≤100 pg/mL	184 (70.8%)	12 (30.0%)	2 (16.7%)	<0.001	77 (72.6%)	100 (64.5%)	21 (41.2%)	0.001
>100 pg/mL	76 (29.2%)***	28 (70.0%)^†††^	10 (83.3%)^†††^		29 (27.4%)***	55 (35.5%)***	30 (58.8%)^†††^	

**NOTE:** Values are medians and interquartile range.

*p value < 0.05 based on the preanhepatic phase value

**p value < 0.01 based on the preanhepatic phase value

***p value < 0.001 based on the preanhepatic phase value

^†^*p* value < 0.05 based on the normal group value

^††^*p* value < 0.01 based on the normal group value

^†††^*p* value < 0.001 based on the normal group value

### Comparison of postoperative outcomes between the 2016 ASE/EACVI and 2009 ASE/EAE recommendations

Patients with diastolic dysfunction (2016 recommendations) remained in the ICU longer and had a higher incidence of overt HFrEF than those with normal diastolic function (Table 5). The proportion of patients using mechanical ventilation was higher in the 2016 diastolic dysfunction group than in the normal group, and the proportion of patients who underwent CRRT was higher in the 2016 indeterminate and dysfunction groups than in the normal group. Patients with diastolic dysfunction (2009 recommendations) also suffered from more frequent development of the HFrEF than those with normal diastolic function. Five patients developed overt HFrEF during the follow-up period. In the 2016 recommendations, four patients in the dysfunction group (33.0%) and one patient in the indeterminate group (2.5%) experienced HFrEF. In the 2009 recommendations, three patients in the dysfunction group (5.9%) and two patients in the indeterminate group (1.3%) experienced HFrEF. However, other outcomes (i.e., total hospital stay, and the development of EAD and AKI) were comparable among the three groups according to the 2019 and 2009 recommendations.

**Table 5:** Comparison of postoperative outcome between the normal diastolic, indeterminate, and diastolic dysfunction groups using the 2016 and 2009 recommendations in patients undergoing living-donor liver transplantation.

	2016 recommendations				2009 recommendations			
Diastolic function group	Normal	Indeterminate	Dysfunction	*p*	Normal	Indeterminate	Dysfunction	*p*
n = 312	260 (83.3%)	40 (12.8%)	12 (3.8%)		106 (34.0%)	155 (49.7%)	51 (16.3%)	
Total hospital stay (day)	25 (21–36)	22 (21–36)	29 (23–42)	0.14	24 (21–37)	25 (21–36)	27 (21–36)	0.817
Total intensive care unit stay (day)	7 (6–7)	7 (6–7)	8 (7–9)^††^	0.014	7 (6–7)	7 (6–7)	7 (6–8)	0.411
Overt HFrEF during follow-up period	0 (0.0%)	1 (2.5%)	4 (33.0%)^†††^	<0.001	0 (0.0%)	2 (1.3%)	3 (5.9%)^†^	0.021
Mechanical ventilation in ICU	106 (40.8%)	20 (50.0%)	12 (100.0%)^†††^	<0.001	43 (40.6%)	66 (42.6%)	29 (56.9%)	0.132
CRRT in ICU	16 (6.2%)	7 (17.5)^†^	3 (25.0%)^†^	0.006	6 (5.7%)	13 (8.4%)	7 (13.7%)	0.231
Early allograft dysfunction during the first week	26 (10.0%)	3 (7.5%)	2 (16.7%)	0.646	5 (4.7%)	20 (12.9%)	6 (11.8%)	0.084
Acute kidney injury during the first week	26 (10.0%)	4 (10.0%)	2 (16.7%)	0.757	12 (11.3%)	13 (8.4%)	7 (13.7%)	0.5

**Abbreviation:** HFrEF, heart failure reduced ejection fraction; ICU, intensive care unit; CRRT, continuous renal replacement therapy

**NOTE:** Values are medians (interquartile range) and numbers (proportion).

^†^*p* value < 0.05 based on the normal group value

^††^*p* value < 0.01 based on the normal group value

^†††^*p* value < 0.001 based on the normal group value

**Fig 2:**
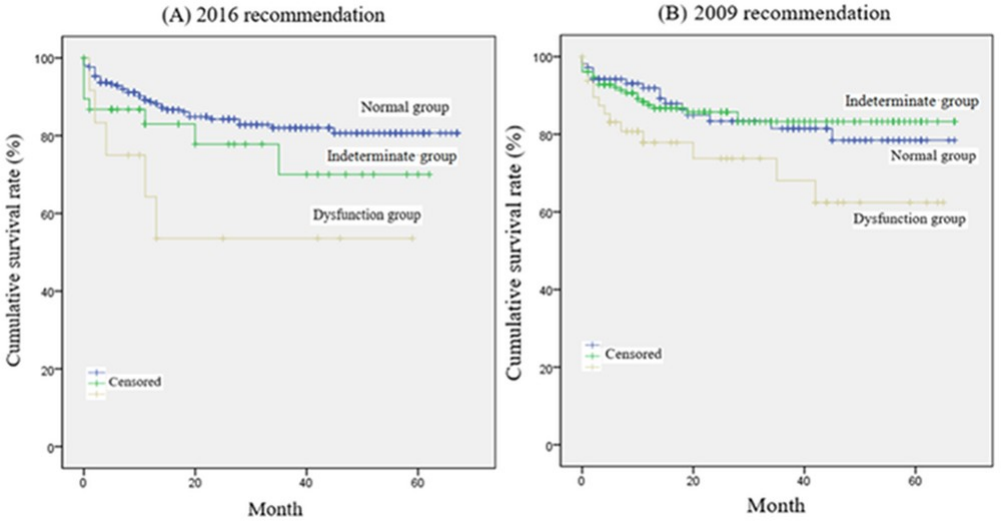
Comparison of the overall patient survival rate between normal diastolic, indeterminate, and diastolic dysfunction groups according to (A) the 2016 recommendations and (B) the 2009 recommendations in living-donor liver transplantation. (A) According to the 2016 recommendations, overall patient survival was significantly different between the normal and dysfunction groups (*p* = 0.007) but did not differ between the normal and indeterminate groups (*p* = 0.183) or between the indeterminate and dysfunction groups (*p* = 0.223). The 1-, 3- and 5-year survival rates were 98%, 93%, and 91% in the normal group; 87%, 70%, and 70% in the indeterminate group; and 64%, 54%, and 54% in the dysfunction group, respectively. (B) According to the 2009 recommendations, overall patient survival was significantly different between the normal and dysfunction groups (*p* = 0.042) and between the indeterminate and dysfunction groups (*p* = 0.027), but not between the normal and indeterminate groups (*p* = 0.836). The 1-, 3-, and 5-year survival rates were 94%, 92%, and 83% in the normal group; 95%, 91%, and 87% in the indeterminate group; and 83%, 74%, and 62% in the dysfunction group, respectively.

According to the 2016 recommendations, the survival rate was better in the normal group than in the dysfunction group; however, no differences in survival rates were observed in the indeterminate group compared with the normal and dysfunction groups (Fig 2). According to the 2009 recommendations, the survival rate was better in the normal and indeterminate groups than in the dysfunction group; however, no difference in survival rate was detected between the normal and indeterminate groups. The all-cause mortalities in our study were as follows: graft insufficiency in 16 patients (5.1%); HCC recurrence in 17 patients (5.4%); infection in 17 patients (5.4%); overt heart failure in 5 patients (1.6%); and coronary artery disease in 1 patient (0.3%). In the 2016 diastolic dysfunction group, five patients died (i.e., overt heart failure in four patients and infection in one patient), after surgery. In the 2009 diastolic dysfunction group, 15 patients died due to overt heart failure (3 patients), acute coronary artery disease (1 patient), infection (5 patients), graft insufficiency (3 patients), and HCC recurrence (3 patients) after surgery.

## Discussion

The main findings of this study were that there was a predominant difference in the prevalence rate of diastolic dysfunction between the 2016 and 2009 recommendations in patients undergoing LDLT. The prevalence rates of diastolic dysfunction and indeterminate diastolic function were lower according to the 2016 recommendations than the 2009 recommendations. The level of concordance between the 2016 and 2009 recommendations was poor. The proportion of patients with a high BNP level (> 100 pg/mL) during surgery decreased significantly in the normal and indeterminate groups according to the 2009 recommendations; however, only the normal group showed an intraoperative decrease in the proportion according to the 2016 recommendations. Patients with diastolic dysfunction had a worse overall-survival rate than those with normal function according to both recommendations. However, there was a difference in the survival rate in the indeterminate group between the 2016 and 2009 recommendations. In the 2009 recommendations, there was a significant difference in patient survival rate between the dysfunction and indeterminate groups; however, the difference was not significant according to the 2016 recommendations.

The 2016 ASE/EACVI recommendations were proposed to simply identify the patients at risk of diastolic dysfunction using TTE findings in a daily clinical setting [20]. However, in previous studies based on general community populations, poor concordance rates of diastolic function between the 2016 and 2009 recommendations were reported that lowered the prevalence of diastolic dysfunction (i.e., only 1.3% by Huttin *et al*. and 1.4% by Almeida *et al*.) in the 2016 recommendations than the prevalence determined by the 2009 recommendations [22,23]. This finding seemed to be largely shared with our result of lower prevalence of diastolic dysfunction in the 2016 recommendations (i.e., 3.8%) than in the 2009 recommendations (i.e., 16.3%). The potential reason for this discrepancy may be related to the inclusion of a new diastolic parameter, such as a TR velocity > 2.8 m/s. TR velocity is a marker of acute or chronic pressure overload, because pulmonary systolic hypertension, derived from the TR velocity, is closely related to overt pulmonary hypertension in patients with diastolic dysfunction [36]. TR velocity may eventually be deemed to play an important role as it represents a more aggravated degree of diastolic function. In our study (2016 recommendations), the total prevalence of patients with a TR velocity > 2.8 m/s was 8.7%, and the proportion for TR velocity > 2.8 m/s was significantly higher in the indeterminate and dysfunction groups than in the normal group. Including TR velocity may have affected the increase in specificity to diagnose diastolic dysfunction in the 2016 recommendations than in the 2009 recommendations. Compared with the previous general population studies [22,23], our prevalence of diastolic dysfunction appeared to be higher, possibly because our study populations suffered from ESLD, which caused pathophysiological changes in cardiac function or geometry [37]. In line with previous work, our study found that a higher MELD score, which represents the severity of hepatic decompensation, was correlated with the degree of diastolic function in both the 2016 and 2009 recommendations [1,6].

BNP is derived from the ventricular wall due to hemodynamic volume or pressure stress and is significantly related to the severity of left ventricular function. Furthermore, a close correlation between serum levels of BNP and diastolic parameters on echocardiography, such as tissue Doppler imaging, is present so that high serum levels of BNP (> 100 pg/mL) are reflected by increased left ventricular filling pressure [38,39]. In previous LT studies, high serum levels of BNP were significantly associated with poor postoperative outcomes, such as graft dysfunction, kidney injury, and patient survival [24,29,40]. In our study, patients with normal diastolic function experienced a significant decrease in the proportion of high BNP level (> 100 pg/mL) through the surgical phases; however, this finding was not evident in the patients with diastolic dysfunction according to both recommendations. Interestingly, there were differences in intraoperative changes in the proportion of patients with a high BNP level in the indeterminate group between the 2016 and 2009 recommendations. Namely, the indeterminate group (2009 recommendations) showed a decrease in the proportion of patients with a high BNP through the surgery, and the proportion of patients with a high BNP level was eventually comparable between the indeterminate and normal groups at the neohepatic phase. However, in the 2016 recommendations, the indeterminate group did not experience a decrease in the proportion of patients with a high BNP level; and the proportion of patients with a high BNP level was higher in the indeterminate group than in the normal group at each surgical phase. These findings potentially explain why only 14 patients (9.0%) in the indeterminate group (2009 recommendations) remained in the indeterminate group (2016 recommendations), and almost all patients (n = 140; 90.3%) in the indeterminate group (2009 recommendations) were reclassified into the normal group (2016 recommendations). One patient in the indeterminate group (2009 recommendations) was reclassified into the dysfunction group (2016 recommendations). Therefore, our results suggest that patients in the indeterminate group (2016 recommendations) may have a higher risk of developing impaired diastolic function than normal function during LDLT compared with the 2009 recommendations. Additionally, patients with indeterminate diastolic findings, defined as 50% of the 2016 diastolic parameters (i.e., average E/e’ > 14, septal e’ velocity < 7 cm/s or lateral e’ velocity < 10 cm/s, TR velocity > 2.8 m/s, and LAVI > 34 mL/m^2^), may require meticulously and continuously monitored cardiac status using ECG or laboratory factors during LDLT.

Cardiovascular complications are a major cause of mortality in patients undergoing LT, including graft rejection and infection that relates to approximately 20% of post-transplant deaths [41]. More than 70% of patients who undergo LT suffer from cardiovascular complications, and approximately 7% of these patients are aggravated to severe heart failure [8]. Preoperative diastolic dysfunction is significantly associated with an increased risk of developing perioperative heart failure and higher rates of 1-year mortality [42,43]. However, successful LT has a positive effect on cardiac functional recovery after LT, as diastolic dysfunction gradually improves during the first year after surgery, together with the cardiac response to stressful stimuli [44–46]. Our study shared previous findings [42,43,47] that patients with diastolic dysfunction (both 2016 and 2009 recommendations) developed an aggravation of cardiac function to overt heart failure and poor overall patient survival than those with normal diastolic function. However, there was a difference in overall patient survival in the indeterminate group between the recommendations. Unlike the 2009 recommendations, there was no difference in the overall survival rate between the dysfunction and indeterminate groups in the 2016 recommendations. The potential explanation is that inclusion of TR velocity > 2.8 m/s played a role to more clearly stratify the mortality risk after surgery. A study by Bushyhead *et al*.[48] suggested that TR (more than a mild degree) is significantly associated with worse patient survival after LT, and another study by Kia *et al*.[49] showed that among various echocardiographic variables, only TR (more than a mild degree) plays a predictive role in patient and graft survival. Ford *et al*.[50] suggested that the cut-off level of TR velocity on 1-year LT mortality was 3.0 m/s, and that backward-pressure, derived from increased and prolonged TR, may cause persistent graft edema related to graft failure and long-term complications. Therefore, TR may be an indicator of hemodynamic load that becomes aggravated when patients are in precarious conditions, such as sepsis [51].

Some limitations of our study should be discussed. First, our study included only patients with a preserved EF (> 50%); therefore, our findings do not apply to patients with a reduced EF. Particularly, because of peripheral vasodilatation, cardiac systolic dysfunction in patients with ESLD is latent at rest [52]. Further study is required to accurately measure systolic dysfunction when patients are challenged during LT. Second, the patients did not routinely undergo post-transplant cardiac function testing using TTE, which was only performed based on the decision of the attending intensive care physician. Eventually, because of the possibility of an under-estimate of the incidence of overt heart failure after surgery, we were unable to investigate patient survival rate related to heart-specific complications, and evaluated all-cause patient survival rate, including graft rejection and infection. A prospective study for heart originating deaths would further clarify the differences between the 2016 and 2009 recommendations. Third, the gold standard to diagnose diastolic dysfunction has not been fully established in clinical settings. Particularly, advanced liver disease causes pathophysiological changes in hemodynamic circulation, such as splanchnic vasodilatation [6]. The diagnostic accuracy of diastolic dysfunction in both recommendations has not been fully demonstrated in patients who underwent LDLT. Finally, because of the small number of patients with diastolic dysfunction in the 2016 recommendations, we were unable to evaluate intraoperative changes in BNP and postoperative outcomes according to the degree of diastolic dysfunction. A further large cohort study is required to clarify the effect of diastolic severity on prognosis in patients who underwent LDLT.

## Conclusions

Clinical application of diastology according to the 2016 ASE/EACVI recommendations in patients who underwent LDLT resulted in a lower prevalence of indeterminate function and overt diastolic dysfunction, and a higher prevalence of normal diastolic function. Therefore, the concordance between previous and current recommendations was poor, which was caused by reclassification of the diastolic functional evaluation. The current 2016 classification may be able to more clearly identify patients at risk for diastolic dysfunction that may result from inclusion of TR velocity > 2.8 m/s. This finding was supported by the intraoperative BNP level, as a diastolic marker. Particularly, patients in the 2016 indeterminate group seem to require an evaluation of cardiac diastolic function more frequently than those in the 2009 indeterminate group, during and after surgery, because the 2016 indeterminate group included ill patients who most likely should be evaluated and treated like the diastolic dysfunction group. Additionally, this finding is not limited to patients undergoing LDLT, but also can be addressed in patients undergoing deceased donor LT [53,54]. The prognostic impact of patients with indeterminate diastolic function and overt diastolic dysfunction needs further investigation in patients who undergo LDLT.

## Supporting information

S1 Table Correlation of MELD score with diastology in 2016 and 2009 recommendations. (DOCX)

S2 Table Comparison of diastology in the 2016 and 2009 recommendation between MELD score ≤ and > 16 points. (DOCX)

## Abbreviations

AKI: acute kidney injury
ASE/EACVI: American Society of Echocardiography/European Association of Cardiovascular Imaging
ASE/EAE: American Society of Echocardiography /European Association of Echocardiography
BNP: brain natriuretic peptide
DM: diabetes mellitus
EAD: early allograft dysfunction
EF: ejection fraction
ESLD: end-stage liver disease
FFP: fresh frozen plasma
HBP: hypertension
HFpEF: heart failure preserved ejection fraction
HFrEF: heart failure reduced ejection fraction
LA: left atrium
LAVI: left atrial volume index
LDLT: living donor liver transplantation
LT: liver transplantation
MELD: model for end-stage liver disease
PRBC: packed red blood cell
TR: tricuspid regurgitation
TTE: transthoracic echocardiography

## References

[1] Yang YY, Lin HC. The heart: pathophysiology and clinical implications of cirrhotic cardiomyopathy. J Chin Med Assoc. 2012; 75:619–623. 10.1016/j.jcma.2012.08.015 23245476

[2] Nagueh SF. Prognostic power of mitral annulus indices of left ventricular diastolic function. J Am Heart Assoc. 2014; 3:e001012 10.1161/jaha.114.001012 24895165 PMC4309106

[3] Kane GC, Karon BL, Mahoney DW, Redfield MM, Roger VL, Burnett JC Jr., et al Progression of left ventricular diastolic dysfunction and risk of heart failure. Jama. 2011; 306:856–863. 10.1001/jama.2011.1201 21862747 PMC3269764

[4] Kuznetsova T, Thijs L, Knez J, Herbots L, Zhang Z, Staessen JA. Prognostic value of left ventricular diastolic dysfunction in a general population. J Am Heart Assoc. 2014; 3:e000789 10.1161/jaha.114.000789 24780207 PMC4309065

[5] Aljaroudi W, Alraies MC, Halley C, Rodriguez L, Grimm RA, Thomas JD, et al Impact of progression of diastolic dysfunction on mortality in patients with normal ejection fraction. Circulation. 2012; 125:782–788. 10.1161/circulationaha.111.066423 22261198

[6] Liu H, Jayakumar S, Traboulsi M, Lee SS. Cirrhotic cardiomyopathy: Implications for liver transplantation. Liver Transpl. 2017; 23:826–835. 10.1002/lt.24768 28407402

[7] Kazankov K, Holland-Fischer P, Andersen NH, Torp P, Sloth E, Aagaard NK, et al Resting myocardial dysfunction in cirrhosis quantified by tissue Doppler imaging. Liver Int. 2011; 31:534–540. 10.1111/j.1478-3231.2011.02468.x 21382164

[8] Eimer MJ, Wright JM, Wang EC, Kulik L, Blei A, Flamm S, et al Frequency and significance of acute heart failure following liver transplantation. Am J Cardiol. 2008; 101:242–244. 10.1016/j.amjcard.2007.08.056 18178414

[9] Dalia AA, Flores A, Chitilian H, Fitzsimons MG. A Comprehensive Review of Transesophageal Echocardiography During Orthotopic Liver Transplantation. J Cardiothorac Vasc Anesth. 2018; 32:1815–1824. 10.1053/j.jvca.2018.02.033 29573952

[10] Donovan RJ, Choi C, Ali A, Heuman DM, Fuchs M, Bavry AA, et al Perioperative Cardiovascular Evaluation for Orthotopic Liver Transplantation. Dig Dis Sci. 2017; 62:26–34. 10.1007/s10620-016-4371-3 27830409

[11] Edvardsen T, Smiseth OA. Evaluation of diastolic function by echocardiography: important progression, but issues to be resolved. Eur Heart J Cardiovasc Imaging. 2018; 19:387–388. 10.1093/ehjci/jex319 29236972

[12] Garg A, Armstrong WF. Echocardiography in liver transplant candidates. JACC Cardiovasc Imaging. 2013; 6:105–119. 10.1016/j.jcmg.2012.11.002 23328568

[13] Lee SG, Moon DB, Hwang S, Ahn CS, Kim KH, Song GW, et al Liver transplantation in Korea: past, present, and future. Transplant Proc. 2015; 47:705–708. 10.1016/j.transproceed.2015.02.015 25891715

[14] Sauer P, Schemmer P, Uhl W, Encke J. Living-donor liver transplantation: evaluation of donor and recipient. Nephrol Dial Transplant. 2004; 19 Suppl 4:iv11–15. 10.1093/ndt/gfh1035 15240843

[15] Don-Wauchope AC, McKelvie RS. Evidence based application of BNP/NT-proBNP testing in heart failure. Clin Biochem. 2015; 48:236–246. 10.1016/j.clinbiochem.2014.11.002 25448029

[16] Grewal J, McKelvie R, Lonn E, Tait P, Carlsson J, Gianni M, et al BNP and NT-proBNP predict echocardiographic severity of diastolic dysfunction. Eur J Heart Fail. 2008; 10:252–259. 10.1016/j.ejheart.2008.01.017 18331967

[17] Richards M, Troughton RW. NT-proBNP in heart failure: therapy decisions and monitoring. Eur J Heart Fail. 2004; 6:351–354. 10.1016/j.ejheart.2004.01.003 14987587

[18] Moller S, Henriksen JH. Cirrhotic cardiomyopathy. J Hepatol. 2010; 53:179–190. 10.1016/j.jhep.2010.02.023 20462649

[19] Saner FH, Neumann T, Canbay A, Treckmann JW, Hartmann M, Goerlinger K, et al High brain-natriuretic peptide level predicts cirrhotic cardiomyopathy in liver transplant patients. Transpl Int. 2011; 24:425–432. 10.1111/j.1432-2277.2011.01219.x 21276088

[20] Nagueh SF, Smiseth OA, Appleton CP, Byrd BF 3rd, Dokainish H, Edvardsen T, et al Recommendations for the Evaluation of Left Ventricular Diastolic Function by Echocardiography: An Update from the American Society of Echocardiography and the European Association of Cardiovascular Imaging. J Am Soc Echocardiogr. 2016; 29:277–314. 10.1016/j.echo.2016.01.011 27037982

[21] Nagueh SF, Appleton CP, Gillebert TC, Marino PN, Oh JK, Smiseth OA, et al Recommendations for the evaluation of left ventricular diastolic function by echocardiography. Eur J Echocardiogr. 2009; 10:165–193. 10.1093/ejechocard/jep007 19270053

[22] Almeida JG, Fontes-Carvalho R, Sampaio F, Ribeiro J, Bettencourt P, Flachskampf FA, et al Impact of the 2016 ASE/EACVI recommendations on the prevalence of diastolic dysfunction in the general population. Eur Heart J Cardiovasc Imaging. 2018; 19:380–386. 10.1093/ehjci/jex252 29236978

[23] Huttin O, Fraser AG, Coiro S, Bozec E, Selton-Suty C, Lamiral Z, et al Impact of Changes in Consensus Diagnostic Recommendations on the Echocardiographic Prevalence of Diastolic Dysfunction. J Am Coll Cardiol. 2017; 69:3119–3121. 10.1016/j.jacc.2017.04.039 28641802

[24] Chae MS, Park H, Choi HJ, Park M, Chung HS, Hong SH, et al Role of serum levels of intraoperative brain natriuretic peptide for predicting acute kidney injury in living donor liver transplantation. 2018; 13:e0209164 10.1371/journal.pone.0209164 30557393 PMC6296541

[25] Hong SH, Kwak JA, Chon JY, Park CS. Prediction of early allograft dysfunction using serum phosphorus level in living donor liver transplantation. Transpl Int. 2013; 26:402–410. 10.1111/tri.12058 23350888

[26] Miller CM, Quintini C, Dhawan A, Durand F, Heimbach JK, Kim-Schluger HL, et al The International Liver Transplantation Society Living Donor Liver Transplant Recipient Guideline. Transplantation. 2017; 101:938–944. 10.1097/tp.0000000000001571 28437386 PMC5642345

[27] Marwick TH, Gillebert TC, Aurigemma G, Chirinos J, Derumeaux G, Galderisi M, et al Recommendations on the Use of Echocardiography in Adult Hypertension: A Report from the European Association of Cardiovascular Imaging (EACVI) and the American Society of Echocardiography (ASE). J Am Soc Echocardiogr. 2015; 28:727–754. 10.1016/j.echo.2015.05.002 26140936

[28] Lang RM, Bierig M, Devereux RB, Flachskampf FA, Foster E, Pellikka PA, et al Recommendations for chamber quantification: a report from the American Society of Echocardiography’s Guidelines and Standards Committee and the Chamber Quantification Writing Group, developed in conjunction with the European Association of Echocardiography, a branch of the European Society of Cardiology. J Am Soc Echocardiogr. 2005; 18:1440–1463. 10.1016/j.echo.2005.10.005 16376782

[29] Chae MS, Koo JM, Park CS. Predictive Role of Intraoperative Serum Brain Natriuretic Peptide for Early Allograft Dysfunction in Living Donor Liver Transplantation. Ann Transplant. 2016; 21:538–549. 10.12659/AOT.899255 27572618

[30] Bernardi M, Calandra S, Colantoni A, Trevisani F, Raimondo ML, Sica G, et al Q-T interval prolongation in cirrhosis: prevalence, relationship with severity, and etiology of the disease and possible pathogenetic factors. Hepatology. 1998; 27:28–34. 10.1002/hep.510270106 9425913

[31] Aggarwal S, Kang Y, Freeman JA, Fortunato FL Jr., Pinsky MR. Postreperfusion syndrome: hypotension after reperfusion of the transplanted liver. J Crit Care. 1993; 8:154–160. 8275160 10.1016/0883-9441(93)90021-c

[32] Bloom MW, Greenberg B, Jaarsma T, Januzzi JL, Lam CSP, Maggioni AP, et al Heart failure with reduced ejection fraction. Nat Rev Dis Primers. 2017; 3:17058 10.1038/nrdp.2017.58 28836616

[33] Pomposelli JJ, Goodrich NP, Emond JC, Humar A, Baker TB, Grant DR, et al Patterns of Early Allograft Dysfunction in Adult Live Donor Liver Transplantation: The A2ALL Experience. Transplantation. 2016; 100:1490–1499. 10.1097/tp.0000000000001240 27326811 PMC4943080

[34] Mehta RL, Kellum JA, Shah SV, Molitoris BA, Ronco C, Warnock DG, et al Acute Kidney Injury Network: report of an initiative to improve outcomes in acute kidney injury. Crit Care. 2007; 11:R31 10.1186/cc5713 17331245 PMC2206446

[35] Conn HO, Leevy CM, Vlahcevic ZR, Rodgers JB, Maddrey WC, Seeff L, et al Comparison of lactulose and neomycin in the treatment of chronic portal-systemic encephalopathy. A double blind controlled trial. Gastroenterology. 1977; 72:573–583. 14049

[36] Lam CS, Roger VL, Rodeheffer RJ, Borlaug BA, Enders FT, Redfield MM. Pulmonary hypertension in heart failure with preserved ejection fraction: a community-based study. J Am Coll Cardiol. 2009; 53:1119–1126. 10.1016/j.jacc.2008.11.051 19324256 PMC2736110

[37] Lee RF, Glenn TK, Lee SS. Cardiac dysfunction in cirrhosis. Best Pract Res Clin Gastroenterol. 2007; 21:125–140. 10.1016/j.bpg.2006.06.003 17223501

[38] Parekh N, Maisel AS. Utility of B-natriuretic peptide in the evaluation of left ventricular diastolic function and diastolic heart failure. Curr Opin Cardiol. 2009; 24:155–160. 10.1097/HCO.0b013e328320d82a 19532102

[39] Mak GS, DeMaria A, Clopton P, Maisel AS. Utility of B-natriuretic peptide in the evaluation of left ventricular diastolic function: comparison with tissue Doppler imaging recordings. Am Heart J. 2004; 148:895–902. 10.1016/j.ahj.2004.02.016 15523324

[40] Toussaint A, Weiss E, Khoy-Ear L, Janny S, Cohen J, Delefosse D, et al Prognostic Value of Preoperative Brain Natriuretic Peptide Serum Levels in Liver Transplantation. Transplantation. 2016; 100:819–824. 10.1097/tp.0000000000001077 26845306

[41] Fouad TR, Abdel-Razek WM, Burak KW, Bain VG, Lee SS. Prediction of cardiac complications after liver transplantation. Transplantation. 2009; 87:763–770. 10.1097/TP.0b013e318198d734 19295324

[42] Zardi EM, Zardi DM, Chin D, Sonnino C, Dobrina A, Abbate A. Cirrhotic cardiomyopathy in the pre- and post-liver transplantation phase. J Cardiol. 2016; 67:125–130. 10.1016/j.jjcc.2015.04.016 26074443

[43] Ruiz-del-Arbol L, Achecar L, Serradilla R, Rodriguez-Gandia MA, Rivero M, Garrido E, et al Diastolic dysfunction is a predictor of poor outcomes in patients with cirrhosis, portal hypertension, and a normal creatinine. Hepatology. 2013; 58:1732–1741. 10.1002/hep.26509 23703953

[44] Torregrosa M, Aguade S, Dos L, Segura R, Gonzalez A, Evangelista A, et al Cardiac alterations in cirrhosis: reversibility after liver transplantation. J Hepatol. 2005; 42:68–74. 10.1016/j.jhep.2004.09.008 15629509

[45] Liu H, Lee SS. What happens to cirrhotic cardiomyopathy after liver transplantation? Hepatology. 2005; 42:1203–1205. 10.1002/hep.20911 16250041

[46] Wong F, Girgrah N, Graba J, Allidina Y, Liu P, Blendis L. The cardiac response to exercise in cirrhosis. Gut. 2001; 49:268–275. 10.1136/gut.49.2.268 11454805 PMC1728392

[47] Karagiannakis DS, Vlachogiannakos J, Anastasiadis G, Vafiadis-Zouboulis I, Ladas SD. Diastolic cardiac dysfunction is a predictor of dismal prognosis in patients with liver cirrhosis. Hepatol Int. 2014; 8:588–594. 10.1007/s12072-014-9544-6 26202764

[48] Bushyhead D, Kirkpatrick JN, Goldberg D. Pretransplant echocardiographic parameters as markers of posttransplant outcomes in liver transplant recipients. Liver Transpl. 2016; 22:316–323. 10.1002/lt.24375 26609681

[49] Kia L, Shah SJ, Wang E, Sharma D, Selvaraj S, Medina C, et al Role of pretransplant echocardiographic evaluation in predicting outcomes following liver transplantation. Am J Transplant. 2013; 13:2395–2401. 10.1111/ajt.12385 23915391

[50] Ford HJ, Aris RM, Andreoni K. Screening for portopulmonary hypertension with transthoracic echocardiography: implications for early mortality associated with liver transplantation. Am J Respir Crit Care Med. 2009; 180:378; author reply 378–379. 10.1164/ajrccm.180.4.378a 19661254

[51] Vallabhajosyula S, Geske JB, Kumar M, Kashyap R, Kashani K, Jentzer JC. Doppler-defined pulmonary hypertension in sepsis and septic shock. J Crit Care. 2018; 50:201–206. 10.1016/j.jcrc.2018.12.008 30553991

[52] Pagourelias ED, Sotiriou P, Papadopoulos CE, Cholongitas E, Giouleme O, Vassilikos V. Left Ventricular Myocardial Mechanics in Cirrhosis: A Speckle Tracking Echocardiographic Study. Echocardiography. 2016; 33:223–232. 10.1111/echo.13010 26174780

[53] VanWagner LB, Harinstein ME, Runo JR, Darling C, Serper M, Hall S, et al Multidisciplinary approach to cardiac and pulmonary vascular disease risk assessment in liver transplantation: An evaluation of the evidence and consensus recommendations. Am J Transplant. 2018; 18:30–42. 10.1111/ajt.14531 28985025 PMC5840800

[54] Vetrugno L, Barbariol F, Baccarani U, Forfori F, Volpicelli G, Della Rocca G. Transesophageal echocardiography in orthotopic liver transplantation: a comprehensive intraoperative monitoring tool. Crit Ultrasound J. 2017; 9:15 10.1186/s13089-017-0067-y 28631103 PMC5476533

