# Supplementary material for: The 2016 ASE/EACVI recommendations may be able to more accurately identify patients at risk for diastolic dysfunction in living donor liver transplantation
Source: PLoS One. 2019 Apr 23;14(4):e0215603. doi: 10.1371/journal.pone.0215603 (PMC6478297; doi:10.1371/journal.pone.0215603)
Supplement: S1 Table — (DOCX) [file pone.0215603.s001.docx]

**Supporting information**

**S1 Table.** Correlation of MELD score with diastology in 2016 and 2009 recommendations

|  | Diastology | |
| --- | --- | --- |
|  | 2016 recommendation | 2009 recommendation |
| MELD score (point) | Coefficient = 0.219^†††^ | Coefficient = 0.180^††^ |
| MELD score > 16 points | Coefficient = 0.223^†††^ | Coefficient = 0.165^††^ |

**Abbreviation:** MELD, Model for end-stage liver disease

^††^*p* value < 0.01

^†††^*p* value < 0.001
