# Supplementary material for: The 2016 ASE/EACVI recommendations may be able to more accurately identify patients at risk for diastolic dysfunction in living donor liver transplantation
Source: PLoS One. 2019 Apr 23;14(4):e0215603. doi: 10.1371/journal.pone.0215603 (PMC6478297; doi:10.1371/journal.pone.0215603)
Supplement: S2 Table — (DOCX) [file pone.0215603.s002.docx]

**Supporting information**

**S2 Table.** Comparison of diastology in the 2016 and 2009 recommendation between MELD score ≤ and > 16 points

|  | Diastology in the 2016 recommendation | | | *p* |
| --- | --- | --- | --- | --- |
|  | Normal  (n = 260) | Indeterminate  (n = 40) | Dysfunction  (n = 12) |  |
| MELD score (≤ 16 points) | 169 (65.0%) | 17 (42.5%) | 2 (16.7%) | <0.001 |
| MELD score (> 16 points) | 91 (35.0%) | 23 (57.5%)^††^ | 10 (83.3%)^††^ |  |
|  | Diastology in the 2009 recommendation | | | *p* |
|  | Normal  (n = 106) | Indeterminate  (n = 155) | Dysfunction  (n = 51) |  |
| MELD score (≤ 16 points) | 76 (71.7%) | 86 (55.5%) | 26 (51.0%) | 0.011 |
| MELD score (> 16 points) | 30 (28.3%) | 69 (44.5%)^††^ | 25 (49.0%)^†^ |  |

**Abbreviation:** MELD, model for end-stage liver disease

^†^*p* value < 0.05 based on the normal group

^††^*p* value < 0.01 based on the normal group

**NOTE:** Values are expressed as number and proportion.
